# Correction: Reduced expression of AMPK-ß1 during tumor progression enhances the oncogenic capacity of advanced ovarian cancer

**DOI:** 10.1186/1476-4598-13-161

**Published:** 2014-08-04

**Authors:** Cuilan Li, Vincent WS Liu, Pui Man Chiu, Kwok-Ming Yao, Hextan YS Ngan, David W Chan

**Affiliations:** 1 Department of Obstetrics & Gynecology, The University of Hong Kong, 6th Floor, Professorial Block, Queen Mary Hospital, Pokfulam, Hong Kong, SAR, People’s Republic of China; 2 Department of Biochemistry, LKS Faculty of Medicine, The University of Hong Kong, Hong Kong, SAR, People’s Republic of China; 3 Department of Obstetrics & Gynecology of The Third Affiliated Hospital of Guangzhou Medical University & Key Laboratory for Major Obstetric Diseases of Guangdong Province, Guangzhou, People’s Republic of China

## Correction

After publication of this article [1] the authors noticed an error in Figure 5A, B and D (Figure 1 here). In Figure 5A (Figure 1 here), the same image from AMPK-β1 was erroneously used for pAMPKα of A2780cp- β1 panel. The correct image for pAMPKα is now provided. In Figure 5B and D (Figure 1 here), the panel of OV2008-sh β1 used two different sets of b-actin. The b-actin in Figure 5B (Figure 1 here) is now used for the whole panel of OV2008-sh β1 in Figure 5B and D (Figure 1 here). These errors were unintentionally made during figure preparation and do not in any way alter the results or conclusions of this study. The authors apologize that these errors were not detected earlier.

**Figure 1:**
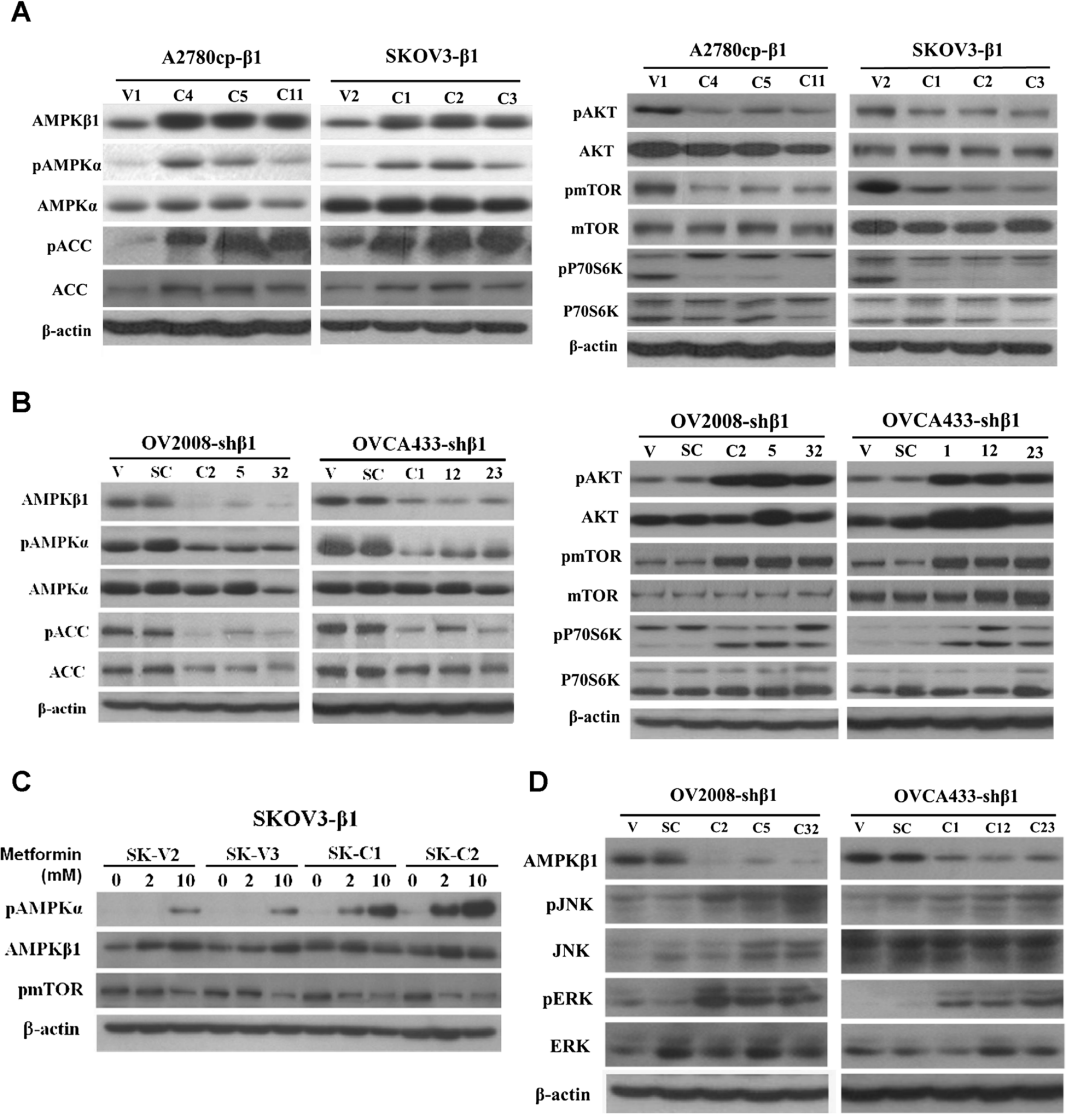
**AMPK-β1 positively regulates AMPK but negatively modulates AKT/mTOR, ERK and JNK activities. (A)** AMPK-β1 overexpression in A2780cp (C4, C5 and C11) and SKOV3 (C1, C2 and C3) cells activated AMPK (increased pAMPKα and pACC (left panel)) but reduced AKT (pAKT) and mTOR (pmTOR and pP70S6K) activities (right panel). **(B)** Knockdown of AMPK-β1 in OV2008 (C2, C5 and C32) and OVCA433 (C1, C12 and C23) cells by shRNA suppressed AMPK activity (decrease of pAMPKα and pACC (left panel)) but elevated AKT (pAKT) and mTOR (pmTOR and pP70S6K) activities (right panel). **(C)** AMPK-β1 overexpression sensitizes ovarian cancer cells to an AMPK activator, metformin, during AMPK activation. SKOV3 cells were treated with the AMPK activator, metformin, at 0-, 2-, and 10-mM concentrations. Stable clones overexpressing AMPK-β1 (C1, C2, C4, and C5) were more sensitive to metformin (2 mM) in the presence of elevated pAMPKα compared with the two empty vector controls (V2 and V3). **(D)** Depletion of AMPK-β1 activates the ERK and JNK pathways, and knockdown of AMPK-β1 in OV2008 (C2, C5 and C32) and OVCA433 (C1, C12 and C23) cells led to an increase in JNK (pJNK) and ERK (pERK) signaling activities.
